# Effect of feeding high oleic soybean oil to finishing pigs on growth performance, carcass characteristics, and meat quality

**DOI:** 10.1093/jas/skae393

**Published:** 2024-12-31

**Authors:** Ashir F Atoo, Crystal L Levesque, Robert Thaler, Keith Underwood, Erin Beyer, Jorge Y Perez-Palencia

**Affiliations:** Department of Animal Science, South Dakota State University, Brookings, SD 57007, USA; Department of Animal Science, South Dakota State University, Brookings, SD 57007, USA; Department of Animal Science, South Dakota State University, Brookings, SD 57007, USA; Department of Animal Science, South Dakota State University, Brookings, SD 57007, USA; Department of Animal Science, North Dakota State University, Fargo, ND 58102, USA; Department of Animal Science, South Dakota State University, Brookings, SD 57007, USA

**Keywords:** carcass characteristics, dietary fat, fatty acids, oleic acid, pork quality, soybean oil

## Abstract

This study aimed to evaluate the effects of dietary fat source and feeding duration on growth performance, carcass characteristics, and meat quality of finishing pigs. A total of 450, 21-wk-old finishing pigs with an average body weight of 113.7 ± 8 kg were housed in 90 pens assigned to one of five dietary treatments in a 2 × 2 + 1 factorial design. Dietary treatments consisted of two fat sources (**CWG**: 4% inclusion of choice white grease and **HOSO**: 4% inclusion of high oleic soybean oil) each provided 2 or 4 wk before marketing. The “+1” diet was corn-based without fat inclusion (**CON**). Observations included growth performance, carcass characteristics, fatty acid (**FA**) profile, and sensory evaluation. Data was analyzed using PROC MIXED in SAS considering dietary treatment as a main effect, feeding duration, and their interactions. Preplanned contrasts were used to compare dietary treatments with the control. From day 14 to 28 and the overall experimental period (day 0–28), pigs fed fat-supplemented diets had a greater (*P* < 0.05) average daily gain and gain-to-feed ratio than CON-fed pigs. There was no significant difference (*P* > 0.05) in growth performance when comparing fat sources or feeding periods. Pigs supplemented with either CWG or HOSO showed a tendency to have a greater (*P* < 0.10) belly weight and belly yield, and a lesser (*P* < 0.10) loin yield and loin muscle area when compared with CON pigs. The loin from pigs fed fat sources had greater (*P* < 0.05) oleic acid and eicosenoic acid concentration when compared with CON. When CWG was compared with HOSO, pigs fed CWG had a higher (*P* < 0.05) concentration of palmitic acid and stearic acid, while the HOSO pigs had a higher concentration of oleic acid and linolenic acid in the loin. In the belly, CON had a higher (*P* < 0.05) concentration of palmitic acid and stearic acid compared to HOSO, while pigs fed fat sources had a higher concentration of oleic acid and eicosenoic acid. Bellies from HOSO had higher (*P* < 0.05) oleic acid and α-linolenic acid methyl ester concentrations, while CWG had higher concentrations of g-linolenic acid. For the sensory evaluation, the palatability and acceptability of pork were not affected (*P* > 0.05) by dietary treatments. In conclusion, supplementation with HOSO not only improved performance but tended to improve some carcass characteristics and increased the concentration of oleic acid, and some other unsaturated FA with a concomitant decrease in the concentration of some saturated FA in pork.

## Teaser

The supplementation of high oleic soybean oil in finishing pig diets can improve the growth performance of pigs and increase the concentration of mono-unsaturated fatty acids in pork, particularly increasing oleic acid concentration when included for 2 wk before marketing. This can be done without negatively affecting carcass quality and consumer sensory preferences.

## Introduction

It has been recommended that humans consume less foods high in saturated fatty acids (**SFA**) and substitute these with healthier options richer in mono-unsaturated fatty acids (**MUFA**) and polyunsaturated fatty acids (**PUFA**) (Statovci et al., 2017). According to the American Heart Association, saturated fatty acids (**FAs**) should only make up about 5% of the daily calorie intake of humans; however, animal meat products typically have high SFA contents. Findings indicate that an increased proportion of MUFA (specifically oleic acid) in one’s dietary intake lowers the risk of coronary heart disease (Lopez et al., 2010). According to Lopez et al. (2010), the ameliorative impact of oleic acid is believed to stem from its biochemical mechanisms, which include altering plasma lipid and lipoprotein levels, inhibiting coagulation, enhancing glucose regulation, and mitigating both inflammation and oxidative stress during fasting conditions. Thus, increasing MUFA concentration in animal meat products is relevant for human health (Castellanos and Rodriguez, 2015). Due to this, there is growing interest in strategies to enhance the unsaturated FA profiles of pork products.

Extensive scientific evidence indicates that the lipid composition of pork is primarily influenced by the FA composition of pig diets (Rosenvold and Andersen, 2003). Therefore, manipulating the FA composition of the pig diet can lead to modifications in the FA profile in pork adipose tissue and muscle (Cava et al., 1997). Conventional feeding of cereal-soybean-based swine diets tends to result in a high content of PUFA and an elevated omega 6:omega 3 FAs ratio in the meat (Teye et al., 2006; Apple et al., 2009a; Benz et al., 2011). While some studies suggest that altering the FA composition could enhance pork quality and provide potential health benefits, there is ongoing debate about the necessity of such changes. For example, Beal et al. (2023) have shown that there is mixed and controversial evidence regarding the negative health impacts of saturated fats and noted limitations in many studies, including potential biases and methodological inconsistencies in studies that have shown the negative health impact of SFA. Despite this, the inclusion of fat sources with higher levels of MUFA remains a potential strategy to improve pork quality and modify its FA composition in line with desired nutritional objectives (Dugan et al., 2015; Leikus et al., 2018). Thus, the inclusion of fat sources with higher levels of MUFA is a timely strategy to enhance pork quality and change pork FA composition.

In the diets for growing-finishing pigs, incorporating fat significantly contributes to the overall energy content of the diet (Okrouhlá et al., 2018). There is a wide range of fat sources used in pig diets ranging from animal to plant sources. When compared to vegetable oils, animal fat such as choice white grease (**CWG**); the inedible fat gotten from swine, tallow, and lard are more widely used, are less expensive, and have higher saturated FAs content (Okrouhlá et al., 2018). Vegetable oils like soybean or rapeseed oil are rich in unsaturated FAs, potentially resulting in healthier products for consumers (Jasinska et al., 2017). Vegetable fat sources such as conventional soybean oil are high in PUFA which will then increase the concentration of PUFA in the pork. High levels of PUFA have been reported to negatively affect the technological quality of pork such as reduction in firmness, affecting the processing of meat products (Meier et al., 2021). High levels of PUFA can also increase the lipid oxidation of pork during processing and storage which causes rancidity thereby reducing the shelf life (Rosenvold and Andersen, 2003; Wood et al., 2008). High levels of PUFA in pork may also negatively affect flavor and texture, causing off-flavors and reducing consumer acceptance of processed pork products (Wood et al. 2008; Meier et al., 2021). Also, recently, new soybean varieties have been developed that are characterized by higher levels of oleic acid, producing soybean oil with a high oleic acid content, which can be included in swine diets to enhance the MUFA content of pork and potentially improve growth performance. In comparison to conventional soybean oil, high oleic soybean oil (**HOSO**) contains 47% more oleic acid (75% vs. 51%) and 70% less linoleic acid (7% vs. 23%) (NRC, 2012; United Soybean Board, 2021).

Gaffield et al. (2022a) evaluated the effects of feeding HOSO (2%, 4%, or 6%) on growth performance and carcass characteristics of growing-finishing pigs during the last 14 wk leading up to slaughter. The authors reported improvements in growth performance, including increased feed efficiency for pigs fed 4% and 6% of HOSO. At the same time, carcasses from those pigs were fatter, resulting in a reduced fat-free lean, which could be related to the duration of the supplementation. It was hypothesized that dietary inclusion of HOSO during the late finishing period (2 or 4 wk before marketing) will enhance the FA profile of pork by increasing MUFA without detrimental effects on carcass characteristics. Thus, considering that added oil increases feed cost and potentially impairs carcass characteristics, this work presents a novel approach to include the newly available HOSO in finishing pig diets for a shorter period to improve the FA profile in pork. Therefore, the objective of this study was to evaluate the influence of the feeding duration of HOSO and CWG on growth performance, carcass characteristics, and meat quality of finishing pigs.

## Materials and Methods

The protocol for this experiment was approved by the Institutional Animal Care and Use Committee at South Dakota State University (**IACUC** #2310-005E).

### Animals and experimental design

A total of 450 21-wk-old growing pigs (113.7 ± 8 kg) housed in 90 pens were arranged in a 2 × 2 + 1 factorial design based on fat source (animal fat or HOSO) and feeding duration before marketing (2 or 4 wk). Pens were single-sex with five pigs per pen. The experiment was conducted in the wean-to-finish barn at South Dakota State University Swine Education and Research Facility, located in Brookings, South Dakota. The facility contains two rooms of 49 pens each; 45 pens (1.8 m by 2.4 m) in each room were assigned to dietary treatments, and the remaining pens housed extra pigs and/or used as medical treatment pens where necessary. All pens contained one dry self-feeder and one cup waterer to allow for ad libitum access to feed and water. The facility operates on mechanical ventilation, with the temperature set at 14.4 °C for the entire experimental period. Pens of pigs were assigned to experimental diets containing one of two different fat sources (CWG:CON diet including 4% of CWG and HOSO:CON diet including 4% of HOSO) each provided at one of the two feeding durations (2 or 4 wk before marketing). The levels of fat supplementation are based on a previous study (Gaffield et al., 2022a, 2022b), where 4% supplementation of HOSO was sufficient to influence growth performance and FA profiles balanced against the cost of added fat. The study commenced 4 wk prior to the expected first cut of pigs (marketing) based on historical barn closeout data. The “+1” diet was a corn-based diet without fat inclusion (**CON**) provided for 4 wk representing the “negative” control. Experimental diets began at 4 or 2 wk before marketing according to the feeding duration program. Pens assigned to CWG or HOSO and 2 wk feeding duration were provided CON for 2 wk prior to their respective fat-supplemented diets. Pigs were blocked by sex and body weight (**BW**) (three BW categories: block 1 = 105.46 kg; block 2 = 119.15 kg; and block 3 = 116.28 kg). Each BW group was put on the experiment in 2 wk increments with block 1 started first. This was done to allow heavier pigs to start first because they were the closest to marketing. Diets were formulated to meet or exceed nutrient requirements (NRC, 2012) in a single feeding phase during the experimental period. The lysine:calorie ratio was constant across all diets (Table 1).

**Table 1.** Experimental diets formulation and calculated composition^1^

Ingredient, %	CON	CWG	HOSO
Corn	86.76	82.61	82.62
Soybean meal	11.00	11.00	11.00
L-lysine HCl	0.20	0.26	0.26
Threonine Pro80	0.077	0.121	0.119
DL-methionine	0.000	0.038	0.036
L-tryptophan	0.003	0.012	0.012
Choice white grease	0.00	4.00	0.00
High oleic soybean oil	0.00	0.00	4.00
Monocalcium phosphate	0.55	0.55	0.55
Limestone	0.88	0.88	0.88
Salt	0.20	0.20	0.20
Swine vitamin premix	0.05	0.05	0.05
Swine mineral premix	0.15	0.15	0.15
Swine larvicide	0.13	0.13	0.13
Mixed feed	100.00	100.00	100.00
Calculated composition			
ME, Kcal/kg	3320	3539	3523
Crude protein, %	12	12	12
Crude fiber, %	2.06	1.98	1.98
SID Lys, %	0.61	0.65	0.65
Lys:calorie	1.83	1.83	1.83
SID Arg, %	0.60	0.59	0.59
SID Met, %	0.19	0.22	0.22
SID, Met + Cys, %	0.38	0.41	0.41
SID Thr, %	0.40	0.43	0.42
SID Trp, %	0.11	0.12	0.12
SID Ile, %	0.42	0.41	0.41
SID Leu, %	1.06	1.03	1.03
SID Val, %	0.48	0.47	0.47
SID His, %	0.29	0.29	0.29
SID Phe, %	0.52	0.50	0.50
Calcium, %	0.46	0.46	0.46
Phosphorus, %	0.41	0.40	0.40
ATTD P, %	0.18	0.18	0.18
STTD P, %	0.21	0.21	0.21
Sodium, %	0.11	0.11	0.11
Ca/P	1.14	1.17	1.17

^1^CON: corn-based diet without fat inclusion; CWG: CON diet supplemented with 4% of choice white grease; HOSO: CON diet supplemented with 4% of high oleic soybean oil; SID: standard ileal digestibility; ATTD: apparent total tract digestibility; STTD: standard total tract digestibility.

### Experimental procedures

Daily animal care observations included pig behavior, recording daily room average, high and low temperatures, checking waterers and feeders, and treating pigs if needed. Pigs were treated when they exhibited clinical signs of illness and the treatment dose, product used, date given, pig and pen identification, and reason administered were recorded throughout the experimental period.

### Growth performance

Pigs were weighed at day 0 (4 wk before marketing), 14 (2 wk before marketing), and 28 (marketing) of the experiment. Feed disappearance was measured simultaneously with BW on a pen basis and then average daily gain (**ADG**), average daily feed (**ADFI**), and gain:feed ratio (**G:F**) were determined.

### Carcass characteristics and meat quality

To access carcass characteristics and meat quality, 12 pigs from each dietary treatment (four from each BW category, two barrows and two gilts) were marked, transported, and harvested for individual carcass data collection at South Dakota State University Meat Lab. The pigs were slaughtered in three batches (three kill dates) corresponding to the three BW categories with different start dates as noted above in Supplementary File S1. The selection of pigs was done to be representative by individually weighing pigs on the last day and within each BW category, 2 barrows and 2 gilts close to the average BW were selected. Within each batch, the treatments were balanced, i.e., the same number of pigs per treatment in each batch. Hot carcass weight (**HCW**) was measured immediately after evisceration and carcass yield was calculated by dividing HCW by live weight. After 48 h of chilling, each carcass was ribbed between the 10th and 11th rib and allowed to bloom for 30 min prior to carcass data collection, and all data was collected on the anterior portion of the loin where bisected. Data collected included loin eye area, fat thickness, subjective color (scale 1–6; National Pork Board, Des Moines, IA, USA), marbling (Scale 1–10; National Pork Board), and firmness scores (Scale 1–3; 1 = soft, 3 = firm, National Pork Board), in addition to objective color measurements (L*, a*, b*) taken by a Minolta colorimeter (Konica Minolta Sensing Americas, Inc; Ramsey, NJ, USA). Firmness refers to the firmness of the loin eye where the carcass was ribbed between the 10th and 11th rib. Following carcass data collection, each carcass was fabricated, and data was collected on the belly (IMPS 408), bone-in loin (IMPS 410), and boneless loin (IMPS 413). Belly measurements included weight, belly yield (belly weight as a percentage of HCW), belly without spareribs, length, width, scribeline width, belly depth, the length of the belly from the shoulder end, and a subjective belly flop score [according to the method by Arkfeld et al. (2016) on a scale 1–5; 1 = soft, 5 = firm]. Loin measurements included weight, boneless loin percentage of carcass, boneless loin weight, and boneless loin percentage of carcass. The loin had an adjacent fat cover.

### FA profile

After chilling, carcasses received a crayon ID on the belly and lumbar vertebrae of the right side of the carcass for tracking through fabrication to collect bellies and boneless loin samples for FA profile. For the loin, a 1-inch pork chop was taken from the left side of the carcass between the third and fourth rib. A 2-inch belly subsample was taken from the anterior end of the belly without spareribs. For the loin samples used for FA composition of the longissimus muscle was denuded of subcutaneous fat and samples were analyzed with only intramuscular fat. Samples were vacuum sealed and stored at −20 °C until analysis. Samples were sent to a commercial laboratory (FoodChain ID, VA, USA) for FA profile analysis using the AOCS Official Method Ce 1b-89 (AOCS, 2023) and similar to the methods used by Alencar et al. (2021). Briefly, fat was extracted from loin and belly samples and subsamples underwent direct methylation of free FAs to their FA methyl ester using BF3/methanol by refluxing the mixture at a constant temperature. The resulting FA methyl esters were analyzed by GC/FID to obtain a full FA profile from C6 to C24. Results are expressed as a percentage of total FAs.

### Sensory traits

The remainder of the loin after subsampling for FA analysis was taken for sensory traits evaluation by a consumer sensory panel according to the standard guide for meat quality sensory panels published by the American Meat Science Association (**AMSA**). A total number of 60 loin samples (from the 12 pigs per treatment harvested) were used for this purpose. Consumers were recruited in Fargo, ND 58102, USA (the largest city in North Dakota) via email; the consumer panels were held at North Dakota State University and conducted by North Dakota State University representatives. The consumer panels were composed of 84 consumers that were grouped into 12 panels with a panel consisting of 6, 7, or 8 participants. This was spread across 2 d with up to three consumer panels simultaneously assessing the loin samples at a single time. Each panel was fed loin chops from 5 out of the 60 loin samples. Samples were cooked according to the AMSA sensory guidelines on a George Foreman grill to a peak internal temperature of 71 °C and peak temperatures were recorded for each sample. At the beginning of each panel, the consumers were given water, unsalted crackers, and apple juice, which they used to clean their palates after each sample before tasting the next. Before the evaluation of samples, consumers filled out a basic demographic questionnaire regarding individual and household characteristics. Each consumer was fed one sample from each treatment plus the control (total of five), in random order. Samples were evaluated for juiciness, tenderness, flavor liking, and overall liking on 100-point continuous line scales with zero anchored at extremely tough, extremely dry, and dislike extremely and 100 being extremely tender, extremely juicy, and like extremely. Moreover, consumers evaluated each trait as either acceptable or unacceptable and classified each sample as either “unsatisfactory,” “everyday quality,” “better than everyday quality,” or “premium quality.” Samples were cooked to a peak internal temperature of 71 °C and peak temperatures were recorded. All the consumer data were recorded on Samsung tablets using Qualtrics surveys.

### Economic analysis

The feed cost was obtained from the commercial feed mill that made the experimental diets based on ingredient prices for January to March, 2023. The carcass price was obtained from the commercial packing plant that the pigs were shipped to (Smithfield, Sioux Falls, SD 57103, USA). The carcass gain value was then calculated as the average carcass gain weight multiplied by carcass price. Total feed cost was calculated by multiplying the total feed consumed for the entire 4 wk by the feed cost. The facility cost was determined by multiplying the total feeding days (28 d) by US$0.1/hd/d facility cost, according to Carpenter et al. (2016). Income over feed cost (**IOFC**) was calculated by subtracting the total feed cost from the carcass gain value. The income over feed and facility cost (**IOFFC**) was calculated by subtracting the facility cost from the IOFC.

### Statistical analysis

The UNIVARIATE procedure of SAS was used to confirm the homogeneity of variance and to analyze for outliers. The growth performance, carcass characteristics, FA profile, and economic performance data were analyzed as a 2 × 2 factorial design using the PROC MIXED procedure in SAS. In the model, the main effects of dietary treatment (animal fat and HOSO), feeding duration (2 or 4 wk), and their interactions were tested considering a pen as the experimental unit. Contrast tests were used to compare fat supplementation groups with the control group. Tukey’s adjusted means test was used to detect differences between fat supplementation groups. Sensory data were analyzed as a 2 × 2 + 1 factorial design using SAS (Version 9.4; SAS Inst., Inc., Cary, NC, USA) PROC GLIMMIX. Peak temperature was used as a random effect. The peak temperature was used to remove the variation from cooking. An α of 0.05 was set for a level of significance and tendencies from 0.05 to 0.1.

## Results

The analyzed chemical composition of experimental diets used in the present study corresponded to the targets in the diet formulations and was within the tolerance of normal variance (Table 2). The FA profile of the two fat sources used in this study shows that HOSO contained 81% more oleic acid compared to CWG (Table 3).

**Table 2.** Analyzed chemical composition of experimental diets^1^

Item	CON	HOSO	CWG
Crude protein, %	12.41	12.55	12.74
Dry matter, %	87.39	87.86	87.98
Crude fat, %	3.13	5.01	5.77
Crude fiber, %	1.78	1.78	1.75
Ash, %	3.12	2.85	2.88
Gross energy, Kcal/kg	3,845.35	3,873.55	3,879.28
Total amino acids, %			
Taurine	0.19	0.19	0.19
Hydroxyproline	0.02	0.03	0.04
Aspartic acid	1.05	1.05	1.06
Threonine	0.60	0.53	0.54
Serine	0.50	0.51	0.51
Glutamic acid	2.30	2.36	2.32
Proline	0.81	0.82	0.82
Lanthionine	0.00	0.00	0.00
Glycine	0.48	0.47	0.47
Alanine	0.71	0.72	0.71
Cysteine	0.22	0.22	0.25
Valine	0.59	0.59	0.59
Methionine	0.21	0.22	0.25
Isoleucine	0.52	0.52	0.51
Leucine	1.19	1.25	1.21
Tyrosine	0.37	0.37	0.39
Phenylalanine	0.59	0.61	0.60
Hydroxylysine	0.02	0.02	0.02
Ornithine	0.01	0.01	0.01
Lysine	0.72	0.70	0.73
Histidine	0.32	0.33	0.32
Arginine	0.66	0.64	0.67
Tryptophan	0.10	0.11	0.11
Total AA	12.18	12.27	12.32

^1^CON: corn-based diet without fat inclusion; CWG: CON diet supplemented with 4% of choice white grease; HOSO: CON diet supplemented with 4% of high oleic soybean oil.

**Table 3.** The FA profile of the two fat sources and experimental diets^1^

	Fat source^2^		Experimental diet^3^		
FA (% total fat)	HOSO	CWG	CON	CWG	HOSO
C10:0 | Capric acid	0	0.08	-	-	-
C12:0 | Lauric acid	0	0.07	-	-	-
C14:0 | Myristic acid	0.04	1.31	1.3	0.79	0.13
C15:0 | Pentadecanoic acid	0.03	0.06	0.41	0.11	0.04
C16:0 | Palmitic acid	6.55	24.77	17.51	19.72	12.05
C16:1 | Palmitoleic acid	0.1	2.38	1.57	1.16	0.24
C17:0 | Heptadecanoic acid	0.64	0.38	0.51	0.29	0.36
C18:0 | Stearic acid	3.28	14.24	5.79	6.9	3.19
C18:1 | Oleic acid	77.71	42.82	23.68	27.04	44.66
C18:2 | Linoleic acid	8.25	11.25	31.38	34.18	31.07
C18:3a | Linolenic acid methyl ester (alpha)	1.95	0.4	1.48	1.29	1.74
C20:0 | Eicosanoic acid	0.39	0.22	1.07	0.51	0.4
C20:1 | Eicosenoic acid	0.48	0.89	0.99	0.56	0.43
C20:3 | *cis*-11,14,17-eicosatrienoic acid methyl ester	0	0.31	-	-	-
C20:4 | Arachidonic acid methyl ester	0	0.07	0.3	0.16	0.15
C20:5 | Eicosapentaenoic acid	0	0.17	-	-	-
C21:0 | Docosanoic acid	0	0.58	-	-	-
C22:0 | Behenic 0.35% acid	0.39	0	-	-	-
C23:0 | Tricoanoic acid	0.05	0	0.59	0.27	0
C24:0 | Lignoceric acid methyl ester	0.14	0	1.29	0.66	0.43
Crude fat (W/W%)^4^			3.59	5.02	5.48

^1^FA composition expressed as percent of total fat. The “0” means it was below the detectable limit of the assay, while the “-” means that FA was not analyzed.

^2^HOSO: high oleic soybean oil; CWG: choice white grease.

^3^CON: corn-based diet without fat inclusion; CWG: CON diet supplemented with 4% of choice white grease; HOSO: CON diet supplemented with 4% of high oleic soybean oil.

^4^W/W% = grams per 100 grams of sample.

### Growth performance

No interactions were observed between dietary fat source and period of supplementation on pig growth performance throughout the experimental period (Table 4). From day 0 to 14, pigs fed diets supplemented with fat during 4 wk before marketing had a tendency for improved F:G (*P *= 0.081) when compared to those fed fat-supplemented diets 2 wk before marketing. From day 14 to 28, pigs fed fat-supplemented diets for the last 2 wk had a tendency for greater ADFI (*P* = 0.053) compared to 4-wk-supplemented pigs. Overall (day 0–28), there was no effect (*P* > 0.05) of fat source or period of supplementation on the growth performance of finishing pigs. When compared to the control group, pigs fed fat-supplemented diets had a greater (*P* < 0.05) ADG and feed efficiency from day 14 to 28 and the overall experimental period regardless of the fat source. By comparing the control to each fat source separately, CWG-supplemented pigs had a greater (*P* < 0.05) feed efficiency from day 14 to 28 and the overall experimental period, while HOSO-supplemented pigs had a greater (*P* < 0.05) ADG and feed efficiency in the same two periods.

**Table 4.** Interactive effect of fat source and period of supplementation on growth performance of finishing pigs^1^

Item	CON	CWG		HOSO		SEM	*P*-value					
		2 wk	4 wk	2 wk	4 wk		Fat source	Period	Source*Period	Contrast CON*Fat Source	Contrast CON*CWG	Contrast CON*HOSO
Initial BW, kg	113.7	113.3	113.2	113.1	113.6	0.734	0.858	0.822	0.659	0.981	0.908	0.874
Period, day 0–14												
BW day 14, kg	129.8	129.9	130.4	129.6	130.3	0.931	0.810	0.530	0.957	0.542	0.433	0.742
ADG, kg	1.15	1.18	1.23	1.18	1.18	0.041	0.518	0.483	0.607	0.309	0.265	0.456
ADFI, kg	3.63	3.59	3.55	3.70	3.56	0.076	0.427	0.241	0.539	0.873	0.706	0.935
G:F	0.32	0.33	0.35	0.32	0.33	0.010	0.206	0.114	0.757	0.190	0.120	0.402
Period, day 14–28												
BW day 28, kg	144.3	145.3	145.2	145.6	146.2	1.077	0.540	0.840	0.745	0.174	0.250	0.182
ADG, kg	1.06	1.15	1.12	1.19	1.17	0.041	0.293	0.464	0.897	0.048	0.166	0.026
ADFI, kg	3.72	3.80	3.57	3.69	3.61	0.079	0.666	0.053	0.360	0.495	0.673	0.408
G:F	0.28	0.30	0.31	0.32	0.32	0.012	0.146	0.818	0.763	0.030	0.165	0.010
Period, day 0–28												
ADG, kg	1.09	1.147	1.148	1.170	1.1642	0.028	0.468	0.926	0.901	0.034	0.102	0.026
ADFI, kg	3.67	3.65	3.55	3.70	3.64	0.058	0.247	0.159	0.735	0.699	0.420	0.919
G:F	0.30	0.31	0.32	0.32	0.32	0.007	0.816	0.373	0.836	0.006	0.016	0.008

^1^CON: corn-based diet without fat inclusion; CWG: CON diet supplemented with 4% of choice white grease; HOSO: CON diet supplemented with 4% of high oleic soybean oil.

### Carcass characteristics

There was no (*P* > 0.05) interactive effect between fat source and period, and no effect of fat source and period on the carcass characteristics (Table 5). The control group compared to the pigs fed fat-supplemented diets irrespective of the period of supplementation, tended to have a higher (*P* < 0.1) boneless loin weight, boneless loin percentage of carcass, loin muscle area while the pigs supplemented with fat sources tended to have higher (*P* < 0.1) belly weight, belly percentage of carcass and firmness score. A comparison between the control and CWG shows that the control tended to have higher (*P* < 0.1) loin muscle area and bone-in loin percentage of carcass while pigs supplemented with CWG tended to have higher (*P* < 0.1) belly weight and belly percentage of carcass. When control was compared to HOSO, control tended to have a higher (*P* < 0.1) loin muscle area while HOSO tended to have a higher (*P* < 0.1) firmness score.

**Table 5.** Interactive effect of fat source and period of supplementation on carcass characteristics of finishing pigs^1^

Item	CON	CWG		HOSO		SEM	*P*-value					
		2 wk	4 wk	2 wk	4 wk		Fat source	Period	Source*Period	CON*Fats	CON*CWG	CON*HOSO
Final BW, kg	150.8	151.0	151.0	150.5	150.9	1.392	0.618	0.661	0.922	0.869	0.720	0.954
Hot carcass wt, kg	111.6	111.6	111.7	110.8	111.4	1.217	0.642	0.777	0.813	0.872	0.957	0.728
Carcass yield, %	74.02	73.96	73.59	73.63	73.83	0.415	0.913	0.843	0.493	0.561	0.628	0.564
Belly measurements												
Belly wt, kg	10.08	10.63	10.48	10.24	10.60	0.181	0.455	0.566	0.171	0.072	0.054	0.175
Belly, % of carcass wt	9.07	9.53	9.40	9.28	9.54	0.157	0.721	0.655	0.215	0.069	0.073	0.125
Belly without spare ribs wt, kg	11.45	11.99	11.70	11.52	11.59	0.217	0.187	0.626	0.413	0.396	0.204	0.783
Belly length, cm	78.91	79.79	79.64	80.22	79.76	0.539	0.612	0.571	0.778	0.135	0.241	0.119
Scribe line width, cm	27.20	28.00	27.52	27.51	27.56	0.346	0.526	0.541	0.451	0.314	0.248	0.494
Belly depth, cm	3.94	4.03	4.01	4.14	4.02	0.126	0.615	0.579	0.686	0.493	0.666	0.412
Belly width, cm	25.73	26.12	25.92	25.63	25.82	0.291	0.319	0.985	0.517	0.745	0.528	0.969
Flop sore	3.07	3.16	3.24	3.16	3.24	0.248	1.000	0.738	1.000	0.638	0.668	0.668
Loin measurements												
Bone-in loin wt, kg	15.65	15.19	15.26	15.37	15.18	0.255	0.849	0.813	0.619	0.155	0.167	0.222
Bone-in loin, % of carcass wt	14.04	13.60	13.68	13.89	13.64	0.173	0.464	0.625	0.340	0.112	0.083	0.243
Boneless loin wt, kg	4.58	4.38	4.36	4.41	4.30	0.112	0.862	0.591	0.697	0.089	0.137	0.104
Boneless loin, % of carcass wt	4.11	3.92	3.92	3.99	3.86	0.095	0.955	0.467	0.489	0.095	0.121	0.132
Loin muscle area, cm^2^	58.77	56.20	53.21	55.18	53.83	1.651	0.903	0.196	0.621	0.053	0.084	0.070
Fat thickness, cm	2.86	2.70	2.91	2.74	2.93	0.191	0.870	0.295	0.957	0.845	0.812	0.905
Color score	2.69	2.40	2.77	2.61	2.69	0.213	0.770	0.287	0.496	0.733	0.657	0.859
Marbling score	1.20	1.27	1.35	1.52	1.19	0.147	0.777	0.398	0.163	0.353	0.466	0.332
Firmness score	1.90	2.23	2.28	2.07	2.53	0.184	0.822	0.181	0.264	0.075	0.123	0.086
L*	62.50	63.69	61.67	63.07	60.48	0.884	0.311	0.013	0.749	0.765	0.884	0.490
a*	19.50	18.59	19.37	19.55	19.94	0.653	0.246	0.374	0.761	0.850	0.483	0.721
b*	10.56	10.54	10.61	10.47	10.24	0.331	0.510	0.817	0.650	0.783	0.984	0.602

^1^CON: corn-based diet without fat inclusion; CWG: CON diet supplemented with 4% of choice white grease; HOSO: CON diet supplemented with 4% of high oleic soybean oil.

### FAs profile

There was a tendency for an interactive effect of fat source and period of supplementation on the concentration of palmitic acid (*P* = 0.0807), stearic acid (*P* = 0.0862), oleic acid (*P* = 0.0794), linolenic acid methyl ester (*P* = 0.0501), arachidonic acid methyl ester (*P* = 0.0592), and caprylic acid (0.0805) in the loin (Table 6). These interactions suggest greater benefits on the loin FA profile when HOSO is supplemented at 4 wk before marketing. There was no effect of the period of supplementation on the FA concentration in the loin. The pigs fed diets supplemented with HOSO had 8% less (*P* < 0.05) loin concentration of stearic acid when compared to the control. When control was compared to the pigs fed fat sources irrespective of the period of supplementation, the pigs fed diets containing fat sources had 3% more loin concentration of oleic acid and 9% more eicosenoic acid which was a result of the HOSO treatment which had a 5% improvement (*P* < 0.05) in loin concentration of oleic acid and the CWG group having a 11% more (*P* < 0.05) eicosenoic acid concentration in the loin. No interactions were observed between the fat source and period of supplementation and no effect of period of supplementation on the FA concentration in the belly (Table 7). The HOSO group had 5% and 11% less belly concentration of palmitic acid and stearic acid respectively when compared to the control. Pigs fed diets supplemented with HOSO had a 6% improvement (*P* < 0.05) in oleic acid while the CWG group had a 3% higher oleic acid concentration in the belly when compared to the control. The HOSO group had an improvement (*P* < 0.05) in linolenic acid methyl ester (12%) and eicosenoic acid (8%), while the CWG group had 9% high eicosenoic acid in the belly when compared to the control.

**Table 6.** Interactive effect of fat source and period of supplementation on loin fatty acid profile^1^

Fatty acid, % of total fatty acid^2^	CON	CWG		HOSO		SEM	*P*-value					
		2 wk	4 wk	2 wk	4 wk		Fat source	Period	Source*Period	CON*Fats	CON*CWG	CON*HOSO
C6:0 | Caproic acid	0.01	0.00	0.02	0.00	0.01	0.007	0.239	0.160	0.239	1.000	0.657	0.657
C8:0 | Caprylic acid	0.01	0.01	0.03	0.01	0.01	0.008	0.419	0.419	0.081	0.914	0.692	0.843
C10:0 | Capric acid	0.09	0.08	0.09	0.08	0.08	0.005	0.241	0.613	0.314	0.579	0.949	0.344
C12:0 | Lauric acid	0.08	0.07	0.08	0.08	0.08	0.004	0.631	0.234	0.234	0.549	0.715	0.467
C14:0 | Myristic acid	1.34	1.28	1.39	1.28	1.27	0.046	0.168	0.297	0.185	0.566	1.000	0.297
C15:0 | Pentadecanoic acid	0.04	0.03	0.04	0.03	0.03	0.003	0.317	0.213	0.801	0.308	0.575	0.194
C16:0 | Palmitic acid	25.86	25.60	26.42	25.23	24.40	0.460	0.013	0.990	0.081	0.434	0.819	0.101
C17:0 | Heptadecanoic acid	0.22	0.22	0.22	0.22	0.23	0.011	0.545	0.940	0.649	0.571	0.784	0.447
C18:0 | Stearic acid	14.57	14.53	14.60	13.97	13.05	0.286	0.001	0.149	0.086	0.125	0.911	0.009
C20:0 | Eicosanoic acid	0.25	0.25	0.26	0.25	0.25	0.009	0.673	0.888	0.427	0.658	0.564	0.817
C21:0 | Docosanoic acid	0.47	0.48	0.45	0.46	0.47	0.031	0.968	0.861	0.600	0.865	0.888	0.864
**SFA^4^**	42.94	42.51	43.55	41.57	39.81	0.733	0.001	0.5997	0.045	0.198	0.905	0.016
C14:1 | Myristoleic acid	0.00	0.01	0.01	0.00	0.01	0.003	0.697	0.517	0.697	0.476	0.408	0.636
C16:1 | Palmitoleic acid	2.33	2.28	2.27	2.19	2.12	0.093	0.156	0.730	0.847	0.308	0.708	0.139
C17:1 | *cis*-10-Heptadecenoic acid	0.02	0.02	0.03	0.04	0.07	0.024	0.178	0.363	0.796	0.534	0.988	0.252
C18:1 | Oleic acid	43.10	43.51	43.25	44.58	45.83	0.419	<0.001	0.243	0.079	0.015	0.559	0.000
C20:1 | Eicosenoic acid	0.77	0.81	0.90	0.82	0.83	0.031	0.342	0.157	0.247	0.050	0.032	0.152
C22:1 | Docosenoic acid	0.02	0.04	0.04	0.03	0.02	0.017	0.339	0.810	0.773	0.502	0.315	0.828
**MUFA**	46.24	46.71	46.53	47.70	48.94	0.487	0.001	0.242	0.123	0.026	0.522	0.001
C18:2 | Linoleic acid	10.15	10.10	9.29	10.07	10.45	0.664	0.398	0.740	0.376	0.841	0.621	0.897
C18:3g | g-Linolenic acid	0.02	0.02	0.01	0.01	0.02	0.007	1.000	0.353	0.167	0.915	0.922	0.922
C18:3a | Linolenic acid methyl ester (alpha)	0.38	0.36	0.32	0.40	0.47	0.033	0.003	0.462	0.050	0.700	0.382	0.118
C20:3 | *cis*-11,14,17-Eicosatrienoic acid methyl ester	0.16	0.16	0.16	0.14	0.16	0.017	0.771	0.663	0.595	0.581	0.698	0.535
C20:4 | Arachidonic acid methyl ester	0.04	0.07	0.05	0.05	0.07	0.009	0.671	0.814	0.059	0.595	0.756	0.510
C20:5 | Eicosapentaenoic acid	0.07	0.07	0.07	0.06	0.07	0.009	0.336	0.435	0.491	0.590	0.913	0.383
**PUFA**	10.82	10.78	9.92	10.73	11.25	0.803	0.367	0.813	0.339	0.856	0.616	0.865
MUFA:SFA	1.16	1.12	1.14	1.11	1.12	0.029	0.650	0.641	0.807	0.236	0.369	0.205
PUFA:SFA	0.27	0.24	0.26	0.24	0.27	0.021	0.967	0.142	0.641	0.451	0.502	0.481
MUFA:PUFA	5.16	5.99	4.03	5.45	4.21	0.795	0.801	0.039	0.630	0.787	0.878	0.734
IV^3^	60.06	57.65	60.24	58.19	59.45	1.280	0.919	0.137	0.603	0.412	0.479	0.429

^1^CON: corn-based diet without fat inclusion; CWG: CON diet supplemented with 4% of choice white grease; HOSO: CON diet supplemented with 4% of high oleic soybean oil.

^2^Percentage of total FAs found in the extracted fat from loin samples.

^3^IV (iodine value) calculated from fatty acid composition: IV = [C16:1] × 0.95 + [C18:1] × 0.86 + [C20:1] × 0.785 + [C22:1] × 0.723 + [C18:2] × 1.732 + [C18:3] × 2.616; brackets indicate percentage concentration (AOCS, 1998).

^4^SFA = C6:0 + C8:0 + C10:0 + C12:0 + C14:0 + C15:0 + C16:0 + C18:0 + C20:0 + C21:0; MUFA = C14:1 + C16:1 + C17:1 + C18:1 + C20:1 + C22:1; and PUFA = C18:2 + C18:3g + C18:3a + C20:3 + C20:4 + C20:5.

**Table 7.** Interactive effect of fat source and period of supplementation on belly fatty acid profile^1^

Fatty acid, % of total fatty acid^2^	CON	CWG		HOSO		SEM	*P*-value					
		2 wk	4 wk	2 wk	4 wk		Fat source	Period	Source*Period	CON*Fats	CON*CWG	CON*HOSO
C8:0 | Caprylic acid	0.00	0.00	0.00	0.01	0.00	0.001	0.671	0.671	0.671	0.623	1.000	0.370
C10:0 | Capric acid	0.08	0.07	0.07	0.07	0.07	0.002	0.545	0.545	0.315	0.192	0.153	0.338
C12:0 | Lauric acid	0.07	0.07	0.07	0.07	0.07	0.002	0.473	0.719	0.473	0.260	0.187	0.461
C14:0 | Myristic acid	1.23	1.21	1.23	1.21	1.20	0.034	0.623	0.941	0.623	0.640	0.835	0.518
C15:0 | Pentadecanoic acid	0.04	0.04	0.06	0.04	0.04	0.002	0.566	0.341	0.341	0.938	0.887	0.775
C16:0 | Palmitic acid	23.86	23.29	23.31	22.91	22.59	0.306	0.080	0.627	0.574	0.018	0.141	0.005
C17:0 | Heptadecanoic acid	0.26	0.29	0.25	0.28	0.28	0.014	0.730	0.209	0.7876	0.441	0.573	0.399
C18:0 | Stearic acid	12.75	11.97	12.08	11.39	11.24	0.285	0.017	0.944	0.647	0.010	0.107	0.002
C20:0 | Eicosanoic acid	0.25	0.25	0.24	0.24	0.24	0.007	0.556	0.813	0.556	0.385	0.585	0.298
C21:0 | Docosanoic acid	0.66	0.69	0.69	0.67	0.65	0.023	0.133	0.799	0.611	0.572	0.273	0.944
**SFA^4^**	39.21	37.89	38.00	36.89	36.39	0.58	0.015	0.705	0.548	0.004	0.082	0.001
C14:1 | Myristoleic acid	0.00	0.00	0.00	0.00	0.01	0.002	0.372	0.372	0.372	0.413	0.263	0.708
C16:1 | Palmitoleic acid	2.01	2.11	2.03	2.06	1.93	0.080	0.352	0.185	0.787	0.796	0.535	0.881
C17:1 | cis-10-Heptadecenoic acid	0.03	0.02	0.03	0.03	0.03	0.017	0.864	0.678	0.826	0.784	0.752	0.853
C18:1 | Oleic acid	42.70	43.88	43.66	44.97	45.77	0.395	0.000	0.475	0.203	0.000	0.040	<0.001
C20:1 | Eicosenoic acid	0.78	0.86	0.84	0.86	0.85	0.024	0.716	0.876	0.398	0.021	0.024	0.048
**MUFA**	45.53	46.88	46.56	47.91	48.59	0.460	0.002	0.690	0.263	<0.001	0.039	<0.001
C18:2 | Linoleic acid	14.25	14.21	14.39	14.15	13.93	0.437	0.554	0.967	0.643	0.875	0.922	0.701
C18:3 | g-Linolenic acid	0.05	0.04	0.05	0.03	0.04	0.006	0.012	0.895	0.598	0.952	0.295	0.248
C18:3a | Linolenic acid methyl ester (alpha)	0.55	0.55	0.54	0.59	0.64	0.020	0.001	0.273	0.170	0.220	0.760	0.013
C20:3 | cis-11,14,17-Eicosatrienoic acid methyl ester	0.22	0.23	0.25	0.22	0.21	0.010	0.065	0.732	0.175	0.628	0.213	0.712
C20:4 | Arachidonic acid methyl ester	0.10	0.09	0.10	0.10	0.11	0.006	0.137	0.368	0.651	0.373	0.851	0.153
C20:5 | Eicosapentaenoic acid	0.11	0.11	0.11	0.10	0.10	0.005	0.080	0.593	1.000	0.306	0.828	0.101
**PUFA**	15.26	15.23	15.44	15.20	15.02	0.471	0.638	0.975	0.679	0.934	0.908	0.790
**MUFA:SFA**	1.29	1.25	1.26	1.27	1.21	0.032	0.664	0.392	0.275	0.225	0.351	0.200
**PUFA:SFA**	0.42	0.43	0.40	0.41	0.37	0.016	0.144	0.030	0.713	0.433	0.906	0.192
**MUFA:PUFA**	3.14	2.97	3.21	3.11	3.30	0.099	0.290	0.044	0.780	0.978	0.666	0.629
**IV** ^2^	67.42	67.71	66.54	67.22	64.95	0.799	0.203	0.039	0.499	0.363	0.763	0.176

^1^CON: corn-based diet without fat inclusion; CWG: CON diet supplemented with 4% of choice white grease; HOSO: CON diet supplemented with 4% of high oleic soybean oil.

^2^Percentage of total FAs is found in the extracted fat from belly samples.

^3^IV (iodine value) calculated from fatty acid composition: IV = [C16:1] × 0.95 + [C18:1] × 0.86 + [C20:1] × 0.785 + [C22:1] × 0.723 + [C18:2] × 1.732 + [C18:3] × 2.616; brackets indicate percentage concentration (AOCS, 1998).

^4^SFA = C8:0 + C10:0 + C12:0 + C14:0 + C15:0 + C16:0 + C17:0 + C18:0 + C20:0 + C21:0; MUFA = C14:1 + C16:1 + C17:1 + C18:1 + C20:1; PUFA = C18:2 + C18:3g + C18:3a + C20:3 + C20:4 + C20:5.

### Sensory attributes

The palatability ratings in terms of juiciness, tenderness, flavor, and overall liking were similar across all treatments and periods of supplementation with no interactions (Table 8). Pork chops from control pigs tended to have a higher (*P* = 0.053) juiciness acceptability when compared to those from pigs fed CWG while tenderness, flavor, and overall liking were not affected (*P* > 0.05) by fat source. There was no effect of feeding duration (*P* < 0.05) on the acceptability ratings. The pork from pigs fed CWG tended to have a higher (*P* < 0.089) premium quality rating in comparison to the pork from pigs fed CON. All other quality ratings were not different (*P* > 0.05) across treatments and duration of supplementation.

**Table 8.** The effect of fat source and period of supplementation on consumer sensory panelist sensory evaluation for fresh pork chops^1^

	Fat source					Inclusion period			
Item	CON	CWG	HOSO	SEM	*P*-value^2^	2 wk	4 wk	SEM	*P*-value
Palatability ratings^3^									
Juiciness	61.2	62.0	60.7	4.5	0.94	59.3	63.3	4.4	0.54
Tenderness	61.3	63.0	61.3	3.9	0.86	60	64.3	3.9	0.39
Flavor	66.1	65.8	63.3	2.8	0.44	63.8	65.3	2.8	0.67
Overall liking	65.9	65.8	62.8	3.5	0.53	62.4	66.2	3.5	0.36
Palatability acceptability ratings									
Juiciness acceptability	94.6	83.6	89.0	0.54	0.06	82.3	90.1	0.53	0.02
Tenderness acceptability	91.6	90.7	87.4	0.48	0.49	87.5	90.5	0.48	0.55
Flavor acceptability	97.8	93.9	95.1	0.79	0.4	93	95.9	0.81	0.22
Overall liking acceptability	95.2	91.3	91.5	0.58	0.52	88.8	93.7	0.6	0.14
Quality ratings									
Unsatisfactory quality	4.9	6.6	7.2	0.60	0.79	7.2	6.6	0.6	0.79
Everyday quality	54.2	47.6	53.6	0.28	0.49	52.2	49.2	0.28	0.75
Better than everyday quality	36	30.7	32.3	0.29	0.72	31.2	31.8	0.29	0.75
Premium quality	4.5^b^	13.7^a^	6.4^b^	0.61	0.03	8.4	11.7	0.58	0.17

^1^CON: corn-based diet without fat inclusion; CWG: CON diet supplemented with 4% of choice white grease; HOSO: CON diet supplemented with 4% of high oleic soybean oil.

^2^There was no (*P* > 0.05) interactive effect between fat source and period for sensory parameters. abc – Letters indicate significant differences at treatment level (*P* ≤ 0.05) using Tukey’s means separation test.

^3^Sensory scores: 0 = extremely dry/tough/dislike; 50 = neither dry nor juicy, neither tough nor tender, neither like nor dislike; 100 = extremely juicy/tender/like extremely.

### Economic performance

The CON group had the least cost per ton followed by the CWG treatment with HOSO treatment having the highest feed cost per ton (Supplementary File 2). There was an interactive effect (*P* < 0.05) of fat source and period of supplementation on the total feed cost per pig. Carcass gain value per pig was not affected (*P* > 0.05) by dietary treatments but when CON was compared with HOSO, HOSO had a higher (*P* < 0.05) carcass gain value per pig. The comparison between pigs fed CWG and those fed HOSO shows the pigs fed HOSO having a higher (*P* < 0.05) gain value per pig and having lower IOFC and IOFFC than the pig fed CWG. The IOFC and IOFFC were different(*P* < 0.05) across dietary treatments with CON having the highest (*P* < 0.05) IOFC and IOFFC when compared to the pigs fed fat sources while CWG had higher (*P* < 0.05) IOFC and IOFFC when compared with HOSO.

## Discussion

The present study investigated the impact of feeding two sources of fat to finishing pigs on their growth performance, carcass characteristics, and pork quality in terms of fat composition and sensory attributes. Having in mind the benefits of MUFA and the need for decreased concentration of SFA in pork due to its negative impact on human health, this study considered HOSO which is high in MUFA (specifically oleic acid) and low in PUFA (specifically linoleic acid) with also low concentration of SFA. Thus, it was hypothesized that a short period of dietary HOSO inclusion in finishing pig diets (2 or 4 wk before marketing) could increase the concentration of MUFA in pork while reducing the concentrations of PUFA and SFA when compared to CON and CWG, which is a common source of dietary fat used in swine diets.

Supplementation with fat (irrespective of the fat source) improved ADG and G:F in finishing pigs. Improved feed efficiency (De la Llata et al., 2001; Weber et al., 2006; Stephenson et al., 2016) and somewhat reduced feed intake (Engel et al., 2001; NRC 2012) are not uncommon when growing-finishing pig diets are supplemented with fat. While there was no difference in ADG among the two fat sources, Benz et al. (2011) reported the tendency for pigs fed soybean oil to have a higher ADG compared to those fed CWG. In fact, the inclusion of fat sources is currently used in the swine industry to counter the slower growth rate of finisher pigs in the summer months (Liu et al., 2022). The lack of difference in terms of finisher pig growth performance with added fat (irrespective of the fat source) in this study is supported by De la Llata et al. (2001) who reported no difference in performance in the finisher phase. However, these authors reported an improvement in ADG in all phases when growing-finishing diets were supplemented with fat (CWG). Allee et al. (1971) reported that regardless of the source of fat (corn oil and tallow), fat supplementation increased ADG and G:F. In the current study, the improvement in overall ADG with fat supplementation was mainly a result of greater gain in the pigs fed HOSO. This is most likely due to the presence of more unsaturated fats (predominantly oleic acid) in their diets as the digestibility of fat, and by extension energy digestibility, is influenced by fat composition where increased MUFA in the diet increases fat digestibility (Stahly, 1984; Benz et al., 2011). However, the difference in ADG between HOSO and CWG in this study was numerical and not statistically different even though Benz et al. (2011) reported that the ADG of growing-finishing pigs supplemented with 5% soybean oil tended to be higher than pigs that were fed 5% of CWG in the diet.

Gaffield et al. (2022a) provided different levels of HOSO to growing-finishing pigs for 98 d and observed an improvement in feed efficiency which was due to a reduction in ADFI. Whereas, in this present study, the improvement in feed efficiency was as a result of the higher ADG of the pigs supplemented with fat. It is expected that fat supplementation increases the energy density of the feed therefore pigs will consume less to meet their energy needs but in the current study, feed intake was not different. Even with no difference in feed intake, the pigs fed diets supplemented with fat consumed a higher amount of energy because of the higher energy density which could possibly explain the higher ADG. This agrees with other authors (Apple et al., 2009b; Stephenson et al., 2016) who reported no change in ADFI with the inclusion of fat and confirmed that feed intake is regulated by energy intake. According to the review by Wealleans et al. (2021), fat inclusion in late finishing pigs largely does not change the ADFI, and the increase in feed efficiency with the addition of fat is a result of the increased energy density of the diet, as was observed in the present study.

In this work, feeding finishing pigs diets supplemented with either CWG or HOSO for 4 or 2 wk before marketing did not influence any of the carcass characteristics that were considered. This contradicts reports by Gaffield et al. (2022a), who found an improvement in carcass yield when growing pigs were fed diets supplemented with 4% HOSO for 14 wk before slaughter. Supplementation in Gaffield et al. (2022a) was attributed to the heavier bellies as a result of increased fat deposition and to the period of supplementation; growing-finishing phase until slaughter (98-d supplementation). Other authors (Smith et al., 1999; De la Llata et al., 2001; Weber et al., 2006) have reported that fat supplementation in diets improves HCW and carcass yield. This is contrary to results in the present study as there were no differences in HCW and yield with fat supplementation probably due to the short supplementation period (2 or 4 wk). It should be noted that these above-mentioned studies, including that of Gaffield et al. (2022a), supplemented dietary fat for longer durations lasting from the growing phase to the finishing phase. This indicates that the duration of supplementation is a factor that may influence the pork carcass characteristics, and the duration of supplementation in the current study may not have been sufficient to see an effect on the carcass characteristics. Other studies (Bee et al., 2002; Nuernberg et al., 2005; Guillevic et al., 2009) have similarly reported that different fat sources in growing-finishing pig diets did not differentially affect carcass characteristics.

In the current study, FA profile in the loins and bellies were affected with different fat sources in the diet which confirms that the fat composition of pork is largely influenced by dietary fats (Azain, 2001). The effect of fat source on pork was predominantly seen in the concentrations of palmitic acid, stearic acid, and oleic acid. This agrees with Gaffield et al. (2022b), who found a similar trend in belly adipose tissue when they supplemented graded levels of HOSO and compared with the control DDGS diet. However, HOSO was supplemented for 98 d in contrast with the current study that reported similar improvement in FA profile with only 2 or 4 wk before marketing. The increase in oleic acid accompanied by the reduction in the concentrations of palmitic acid and stearic acid was consistent with previous studies by Miller et al. (1990) when they compared pigs fed a control corn-SBM diet with those fed animal fat or high oleic acid sources. In the same manner, other authors (West and Myer, 1987; Rhee et al., 1988; Shackelford et al., 1990) have reported a similar trend with the supplementation of pig diets with elevated levels of MUFA. The lack of difference in belly and loin firmness may be explained by the lack of difference in linoleic acid content between fat source groups. Linoleic acid is reported to be a key factor influencing fat firmness and bacon quality (Azain, 2001). From the foregoing, it can be stated that feeding 4% of HOSO to finishing pigs successfully increased the concentration of oleic acid in the pork while reducing the concentration of palmitic acid and stearic acid. However, in the present study improvements in FA profile are expressed as a percentage and thus the relative impact of the change in FA profile may be less in low fat, compared to higher fat human edible products. For greater application and translation to practical human nutrition recommendations, future research should present FA data as mg/100 g edible portion or another quantitative unit.

Although in the present study it was hypothesized that feeding HOSO will decrease the concentration of PUFA, the PUFA was not reduced even with HOSO containing less PUFA. This in part explains the no difference in flavor, considering that high levels of PUFA in pork have been reported to negatively affect flavor and other technological attributes of pork (Wood et al., 2008; Meier et al., 2021). Further, because there was no effect of the supplementation period, these benefits can be obtained by supplementing HOSO for only 2 wk before marketing with minimal impact on carcass parameters.

From the sensory evaluation, there were no differences in juiciness, tenderness, and flavor among all treatments; however, pigs fed HOSO for 4 wk were evaluated by consumers to have a higher juiciness acceptability. A similar lack of difference in meat sensory attributes was reported by Gaffield et al. (2022b) with HOSO supplementation and by other authors who supplemented diets with elevated amounts of MUFA (Miller et al., 1990; Myer et al., 1992). According to Navarro et al. (2021), linoleic acid is highly linked to differences in pork flavor; the lack of difference in flavor determined in the current study may be explained by the lack of difference in linoleic acid content of the loin. It may be that the degree of unsaturation in the pork may have been insufficient to result in detectable taste differences and hence no difference in the sensory attributes as it has been reported that taste characteristics are greatly influenced by the degree of unsaturation of FA (Mottram, 1998; Benet et al., 2016; Zhao et al., 2017).

Indeed, supplementing fat in the diets of finishing pigs comes at an added cost. In this present study, it was observed that the diet without any fat supplementation had the least cost compared to the fat-supplemented diets and between the fat-supplemented diets, the CWG diet was cheaper than the HOSO diet. In this case, HOSO was relatively expensive in comparison to CWG because the supply or availability of HOSO has not reached a point where it can be readily bought from retailers at a comparable price for inclusion in the livestock industry. Moving forward as people become more aware of the benefits of HOSO, it may drive the demand for HOSO which will make the product more readily available at a comparable price to other animal fat sources used in swine. In this study, HOSO was obtained from the human market. These differences in the total feed cost per pig as a result of differences in feed cost were seen in the IOFC and IOFCC which take into account the cost of feed and the facility cost.

In conclusion, the present study comparing HOSO supplementation side by side with CWG supplementation or no-fat supplementation showed that the inclusion of HOSO in the diet of finishing pigs improved growth performance and also increased the deposition of oleic acid while reducing the concentration of SFA in the loins and bellies without adversely affecting carcass quality and sensory attributes. In most cases, feeding finishing pigs HOSO 4 wk and 2 wk before slaughter produced a similar effect. Therefore, considering the cost associated with the supplementation of HOSO, 2 wk of supplementation becomes the most preferred.

## Supplementary Material

skae393_suppl_Supplementary_File

## Abbreviations

ADG: average daily gain
CON: control
CWG: choice white grease
FA: fatty acid
G:F: gain to feed
HCW: hot carcass weight
HOSO: high oleic soybean oil
IOFC: income over feed cost
IOFFC: income over feed and facility cost
IV: iodine value
MUFA: mono-unsaturated fatty acids
SFA: saturated fatty acids
