# Supplementary material for: Effect of feeding high oleic soybean oil to finishing pigs on growth performance, carcass characteristics, and meat quality
Source: J Anim Sci. 2024 Dec 31;103:skae393. doi: 10.1093/jas/skae393 (PMC11757699; doi:10.1093/jas/skae393)
Supplement: skae393_suppl_Supplementary_File [file skae393_suppl_supplementary_file.docx]

**Supplemental file 1.** Experimental design.^1^

**
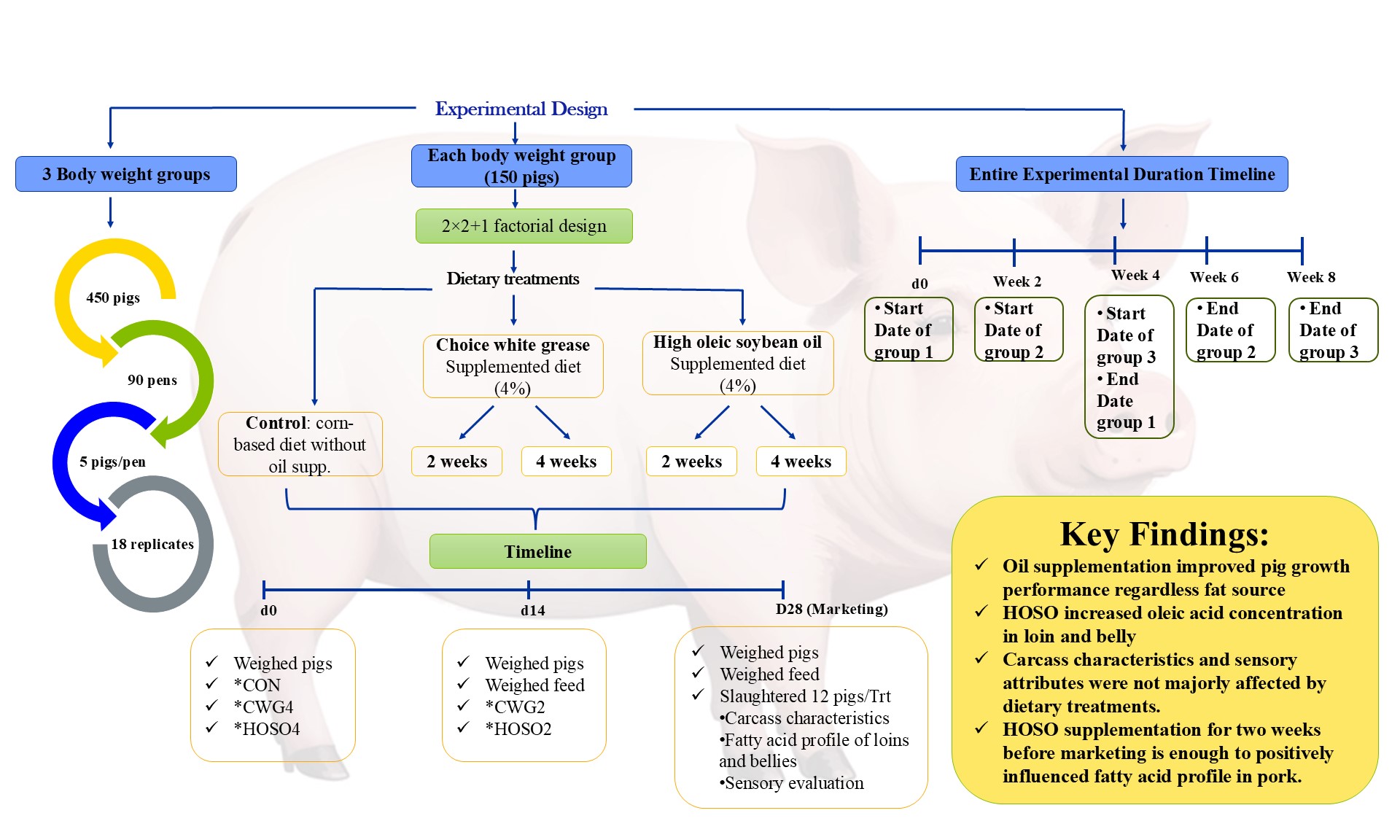
**

^1^CWG: diet supplemented with choice white grease diet; HOSO: diet supplemented with high oleic soybean oil; *CWG4: started feeding CWG for 4 weeks before slaughter; *HOSO4: Started feeding HOSO for 4 weeks before slaughter; *CWG2: started feeding CWG for 2 weeks before slaughter; *HOSO2: Started feeding HOSO for 2 weeks before slaughter; CON4: started feeding CON for 4 weeks before slaughter.

**Supplemental file 2.** Interactive effect of fat source and period of supplementation on the economic performance of finishing pigs.^1^

| **Item** |  | **CWG** | |  | **HOSO** | | **SEM** | **P-value** | | | | | |
| --- | --- | --- | --- | --- | --- | --- | --- | --- | --- | --- | --- | --- | --- |
|  | **CON** | **2 Weeks** | **4 Weeks** |  | **2 Weeks** | **4 Weeks** |  | **Trt** | **Fat source** | **Period** | **CON*FatS** | **CON*CWG** | **CON*HOSO** |
| Feed cost, $ per tonne | 315.69 | 368.07 | 368.07 |  | 435.56 | 435.56 |  |  |  |  |  |  |  |
| Total Feed cost, $ per pig^2,3^ | 36.03^d^ | 38.88^c^ | 40.02^c^ |  | 43.05^b^ | 48.51^a^ | 0.61 | <.0001 | <.0001 | <.0001 | <.0001 | <.0001 | <.0001 |
| Carcass gain value, $ per pig^4^ | 49.22 | 51.59 | 51.41 |  | 52.2 | 52.47 | 1.177 | 0.32 | 0.4772 | 0.9445 | 0.0429 | 0.1162 | 0.0331 |
| Facility cost^5^ | 2.8 | 2.8 | 2.8 |  | 2.8 | 2.8 |  |  |  |  |  |  |  |
| IOFC, $ per pig^6^ | 13.44^a^ | 13.1^a^ | 11.39^ab^ |  | 8.67^b^ | 3.60^c^ | 0.978 | <.0001 | <.0001 | 0.002 | 0.0003 | 0.3311 | <.0001 |
| IOFFC, $ per pig^7^ | 10.64^a^ | 10.30^a^ | 8.59^ab^ |  | 5.87^b^ | 0.80^c^ | 0.978 | <.0001 | <.0001 | 0.002 | 0.0003 | 0.3311 | <.0001 |

^1^CON: corn-based diet without fat inclusion; CWG: CON diet supplemented with 4% of choice white grease; HOSO: CON diet supplemented with 4% of high oleic soybean oil.

^abc^ Within a row means without a common superscript differ (*P*<0.05).

^2^Feed cost × total feed consumption for the 4-week period.

^3^Interaction between fat source and period (*P*=0.0033)

^4^Total carcass gain weight × carcass price.

^5^Facility cost at $0.10/hd per d.

^6^Income over feed cost = carcass gain value – total feed cost.

^7^Income over feed and facility = IOFC – facility cost for 28 days.
